# Fabrication of gold/graphene nanostructures modified ITO electrode as highly sensitive electrochemical detection of Aflatoxin B1

**DOI:** 10.1371/journal.pone.0210652

**Published:** 2019-01-16

**Authors:** Ismail I. Althagafi, Saleh A. Ahmed, Waleed A. El-Said

**Affiliations:** 1 Chemistry Department, Faculty of Applied Science, Umm Al-Qura University, Makkah, Saudi Arabia; 2 Department of Chemistry, Faculty of Science, Assiut University, Assiut, Egypt; Beijing University of Chemical Technology, CHINA

## Abstract

Aflatoxins (AFs) are a family of fungal toxins that produced in food and feed by two Aspergillus species (Aspergillus flavus and Aspergillus parasiticus). Several techniques have been reported for AFs detection including high-pressure liquid chromatography, enzyme-linked immunosorbent assay, surface plasmon resonance and recombinant immune blotting assay. But, these methods are disadvantaged because they consumed a long time for analysis; in addition, they required a piece of complicated and expensive equipment. Therefore, developing of inexpensive sensors with high selectivity and sensitivity for detecting of AFs levels without extensive sample preparation has received great attention. Several electrochemical AFs sensors have been reported; however, there is still a need for developing a new, simple and rapid electrochemical AFs sensor. Here, we have developed a new AFs sensor based on Au nanostructures/graphene nanosheets modified ITO substrate that could enhance the Raman effect and the electrochemical conductivity. The modified electrode was prepared based on layer-by-layer electrochemical deposition method. AFs antibody was immobilized onto the Au nanostructures/graphene nanosheets; then it was used as a probe for rapid, simple and cheap detection of AFs level using Raman spectroscopy and electrochemical techniques. Our results demonstrated that the developed system showed a simple, easy and sensitive sensor for monitoring low concentrations of AFB1 with a detection limit of about 6.9 pg/mL, also it allowed the determination of AFB1 in spiked food samples.

## Introduction

Mycotoxins are one of the most poisonous toxins worldwide; hence, detection of mycotoxins in both foods and feeds is needed to maintain good foods quality and human health. Aflatoxin B1 (AFB1) is a toxin that produced by both Aspergillus flavus and Aspergillus parasiticus **[1]**, which could induce a higher risk of the incidence of hepatocellular carcinomas, hepatitis virus (B & C) and concerned with liver cancer induction **[1]**. AFB1 was classified as Group 1 carcinogen according to the International Agency for Research on Cancer (IARC), it is well known to arise naturally in agricultural products such as peanuts, corn, and animal feeds **[2]**. Therefore, early and rapid detection of AFB1 contaminations in grains and animal feeds is an urgent issue for saving human lives **[3]**. Different tools were reported for quantitative detection of AFs including thin layer chromatography (TLC), high-pressure liquid chromatography (HPLC), enzyme-linked immunosorbent assay (ELISA) and recombinant immune blot assay (RIBA) **[4, 5]**. Although, these assays show high sensitivity but typically require extensive sample preparation, expensive instruments, time-consuming, and the high volumes needed **[6]**, which limited their applications in food products detection. Thus, there is an urgent need for developing a simple and highly sensitive strategy for AFs detection. Sensors have many advantages including high sensitivity, high selectivity, and allowing real-time monitoring of the targets, which are needed for bioanalytical chemistry.

Gold nanoparticles (Au NPs) was considered one of the most widely used metal NPs that could facilitate the electrons transfer and act as tiny conduction centers **[7]**. Several optical and electrical AFB1 sensors have been developed **[8]**. Linting and his group have presented AFB1 electrochemical immunosensor based on graphene/conducting polymer/Au NPs/Au electrode surface with a limit of detection (LOD) of 1 fM **[9]**. Graphene has shown unique electrical, mechanical, thermal, and optical properties, which results in wide applications in many fields such as nanoelectronics, sensors, capacitors and composites **[10]**. Several techniques were reported for the preparation of graphene sheets such as chemical vapor deposition, electrochemical, thermal or chemical reduction of graphene oxide (GO) **[11]**. Electrochemical reduction of GO is a simple, fast and eco-friendly technique. Reduced graphene oxide (rGO) is the material, which results from the incomplete reduction of GO, thus it is considered as an intermediate state between graphene and GO, which contains many functional groups and defects. rGO has great potential uses in many fields including sensors, optical devices, photocatalysts, electrochemical analyses and solar cells **[12]**. The large surface area of rGO makes it a promising matrix for fabrication of rGO/metal NPs hybrids **[13]**, which results in enhancing the metal NPs stability, preventing graphene agglomeration and improved the electrical conductivity of the rGO film. Hence, rGO/metal NPs systems have been reported in various promising applications such as catalysis, energy conversion, surface-enhanced Raman scattering (SERS), chemical/sensors and fuel cells. Various approaches have been reported for fabrication of rGO/metal nanocomposites such as chemical, thermal, microwave and photochemical methods **[14]**. The chemical reduction of GO and metallic precursors with strong reductants is the most common approach, however, it is disadvantaged due to the uses of highly toxic chemicals with long reaction times at high temperature. Recently, electrochemical technique was used for reduction of GO in the presence of gold chloride that results in deposition of both Au and rGO and produce rGO/Au nanocomposite film on the electrode surface, which enhance both conductivity and the surface area of the Au/rGO composite in comparing with the pure graphene film **[15]**. In our previous work, we have developed different modified substrates to enhance their SERS and cyclic voltammetry (CV) responses **[16, 17]**. Here, we have reported the fabrication of Au nanodots/rGO nanosheets/ITO electrode; then we have used it as a highly sensitive and AFB1 sensor for early detection of very low concentrations of AFB1 by using Raman and CV techniques. The principle of this sensor based on monitoring the electrochemical conductivity changes that result from the interaction between AFB1 in the solution and anti-AFB1 immobilized on Au nanodots/rGO nanosheets/ITO electrode. Also, this modified sensor was used to monitor AFB1 molecules in peanut sample spiked with different amounts of AFB1.

## Materials and methods

### 2.1. Materials

Chloroauric acid tetrahydrate (HAuCl_4_⋅4H_2_O) and ITO coated glass slides were obtained from Sigma-Aldrich (3050 Spruce St, St. Louis, MO63103, USA). An aqueous solution of Graphene oxide (1g/L, w/v) was obtained from Graphene Supermarket. Potassium chloride, sodium chloride, methanol, and ethanol were obtained from Sigma. Monosodium phosphate and disodium phosphate were obtained from Merck. Potassium ferricyanide and potassium ferrocyanide were obtained as analytical reagent grade. Aflatoxin B1 antibody was obtained from Sigma-Aldrich. All the aqueous solutions were prepared by using deionized water (DIW) (18.2 MΩ·cm) that purified by a Purite purification system. The buffer system used in this work was phosphate buffer saline (0.20 mol/L) at pH 7.4 that was prepared by dissolving disodium hydrogen phosphate (0.058 g), sodium dihydrogen phosphate (0.01015 g), sodium chloride (0.40 g) and potassium chloride (0.01 g) in 50 mL DIW and then adjusting pH to 7.4 with H_3_PO_4_ or NaOH solutions.

### 2.2. Instruments

All electrochemical measurements were carried out by using micro-Autolab, potentiostat/galvanostat instrument (Metrohm Model 663VA stand) controlled by Autolab Nova software. A handmade three-electrode system consisted of a platinum wire as an auxiliary electrode, Ag/AgCl as a reference electrode, and Au/rGO/ITO (1 cm x 2 cm) as the working electrode was used for the different electrochemical measurements. The surface morphology was analyzed by using a scanning electron microscope (SEM) (JEOL JSM-5400 LV, Japan), the samples were coated with gold film via FINE COAT JEOLJTC-1100E ION SPUTTERING DEVICE at room temperature (RT). Raman spectra were recorded with a Bruker Senterra Raman microscope (Bruker Optics Inc., Germany) with 785 nm excitation, 1200 rulings mm-1 holographic grating and a charge-coupled device (CCD) detector. The acquisition time was 3 sec with a power of 50 mW.

### 2.3. Preparation of gold nanostructures/graphene nanosheets modified ITO electrode

The ITO-coated glass substrates with a geometrical size of 20 mm x10 mm x1.1 mm was cleaned via sequential sonication for 15 min each in 1% Triton X-100, DIW, and ethanol, respectively; then in a basic piranha solution (H_2_O_2_: NH_3_: H_2_O ratio, 1:1:5) for 90 min at RT. Finally, the substrates were cleaned again with DIW and ethanol for 15 min for each; and dried under nitrogen stream. A thin layer of rGO was electrochemically deposed onto the ITO substrate by applying a negative potential of -1.6 V for 10 seconds to a solution of 4 mg/mL of GO in 0.1 M of sodium sulfate as an electrolyte. Then, rGO nanosheets/ITO substrate was then decorated with Au nanostructures as following: Au nanodots electrochemical deposed from an aqueous solution of 1 mM HAuCl_4_ by applying a negative potential of -0.9 V against Ag/AgCl electrode for 30 seconds. The surface morphologies were analyzed with SEM.

### 2.4. Preparation of AFB1 aflatoxins-sensors

The AFB1 antibody (anti-AFB1) solution in phosphate buffered saline (PBS) (10 mM, pH 7.4) have immobilized onto the modified substrate based on self-assembly technique for 6 h at 4°C. Then, the substrate was rinsed with PBS solution. The immobilization of anti-AFB1 was confirmed by using CV technique.

### 2.5. Preparation of food and spiking

To obtain representative samples of naturally contaminated foodstuffs, peanut was spiked with AFB1. Typically, peanut samples were incubated with different concentrations of AFB1 in absolute methanol. Then, the spiked samples were vortexed and dried overnight in the fume hood at RT.

### 2.6. Extraction of aflatoxins from food samples

Each spiked aliquot sample was extracted by vortex the samples with 1.5 ml of methanol/water (3:1, v/v) mixture and then by shaking for 2 hours at RT. The spiked peanut aliquot sample was centrifuged at 4000 rpm for 5 min and then collected the resulting supernatant.

### 2.7. Detection of aflatoxins

AFB1 was monitored by recording changes in the redox current response, and Raman spectroscopy of the AFs-Ab adsorbed on Au/graphene/ITO. The Raman spectra and CV techniques were used to monitor the presence and quantitative detection of AFB1.

## Results and discussion

### 3.1. Fabrication and characterization of gold nanostructures/rGO nanosheets modified ITO electrode

**Fig 1:**
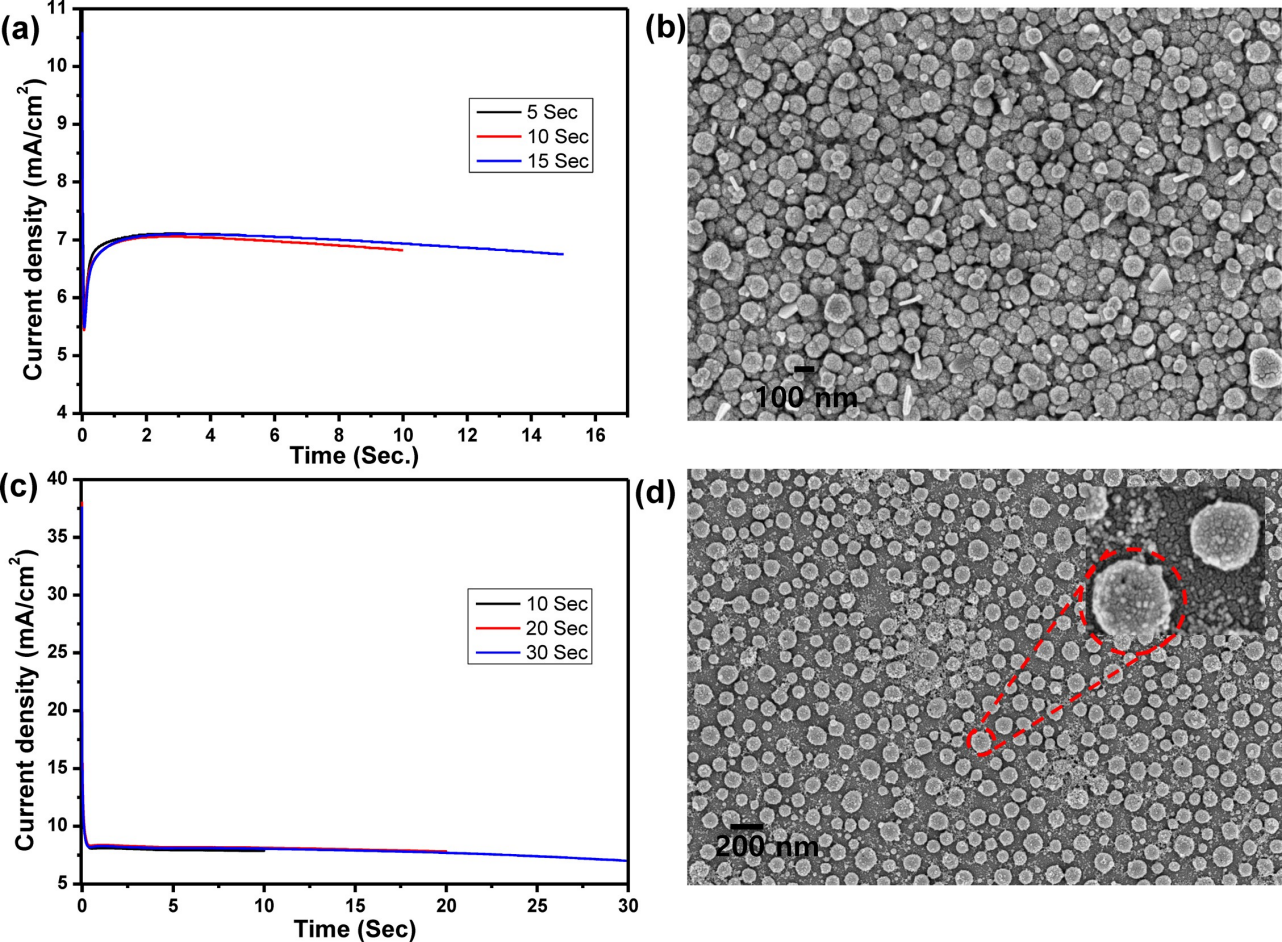
(a) i-t curves for electrochemical deposition and reduction of GO for different periods (5, 10 and 15 sec), (b) SEM image for rGO nanosheets modified ITO electrode that formed after electrochemical deposition for 15 sec, (c) i-t curves for electrochemical deposition of Au NPs onto rGO/ITO electrode for different periods (10, 20 and 30 sec), and (d) SEM image for Au nanodots modified rGO/ITO electrode after 30 sec.

**Fig 2:**
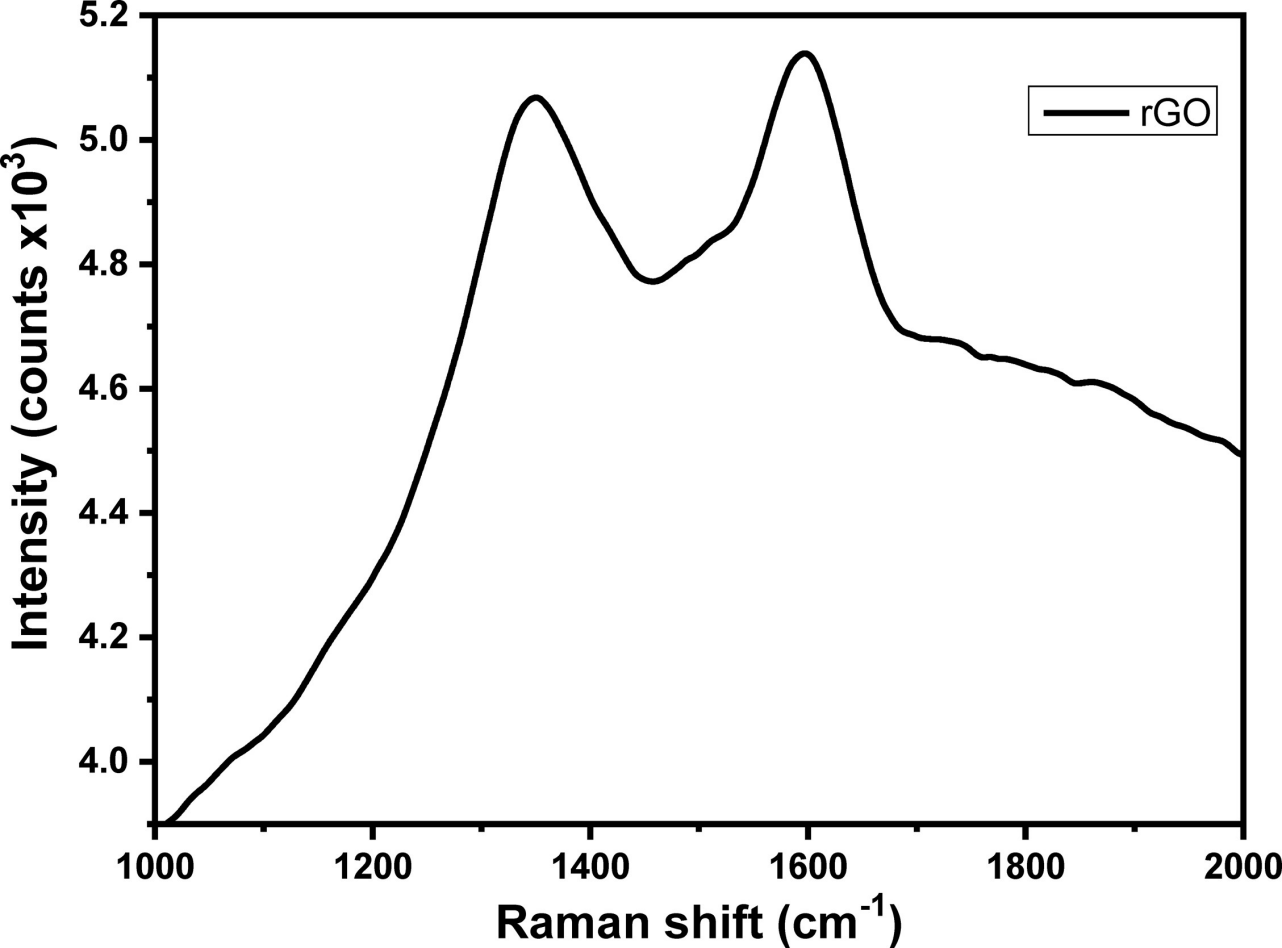
Raman spectrum of rGO nanosheets modified ITO electrode.

In the present work, Au nanodots/rGO nanosheets/ITO modified electrode has been synthesized based on layer-by-layer electrochemical deposition technique. GO dispersion solution was prepared and used as an electrolyte for fabrication of a layer of rGO on ITO surface based electrochemical deposition method by applying a cathodic potential for different deposition time. **Fig 1A** demonstrates the current–time (i–t) transient curves during the deposition processes at a potential of −1.6 V for different deposition time (5, 10, and 15 sec), which illustrates that the current density was dramatically decreased until reaches the minimum of 0.05 sec and then increased to reach the stationary value at 0.2 sec; the charge transfer after 15 sec was about 1.9x10^-3^ coulomb. The topographic SEM image of the rGO film modified ITO surface was represented in **Fig 1B**, which clearly illustrated the fabrication of an array of rGO nanosheets with circular-like structure morphology and a diameter of about 100 nm. Then, rGO nanosheets were decorated with Au nanodots based on electrochemical deposition of Au from Au^3+^ aqueous solution. **Fig 1C** demonstrates the i–t transient curves during the deposition of Au nanodots at a potential of −0.9 V for different deposition time (10, 20 and 30 sec), which illustrates that the current density was first decreased and then increased and again decreased to a stationary value after 0.6 sec; the charge transfer was about 2.1 x10^-3^ coulomb after 30 sec. The current transient profile demonstrates the initial nucleation and growth process during metal deposition. SEM image of the Au nanodots/rGO nanosheets film modified ITO electrode (**Fig 1D**) showed the formation of Au nanodots on the rGO nanosheets with a diameter of about 10 nm. The reduction of GO during the deposition process was confirmed based on the Raman technique. For Raman measurement, the ITO electrode was immersed in a solution of 4 mg/mL of GO in 0.1 M of sodium sulfate and applying a negative potential of -1.6 V for 10 seconds. Then, the rGO modified electrode was rinsed with DIW and dried under N_2_ gas; and finally, the modified substrate was used to measure the Raman spectrum. **Fig 2** represented Raman spectrum of a layer of rGO deposited into ITO electrode by using a 635 nm excitation laser, which demonstrated the appearance of D and G bands at 1351 and 1558 cm^-1^, respectively. The I_D_/I_G_ is about 1.03, which confirmed the formation of rGO film on the ITO substrate [18].

### 3.2 Electrochemical characterization of rGO/Au/ITO electrode

**Fig 3:**
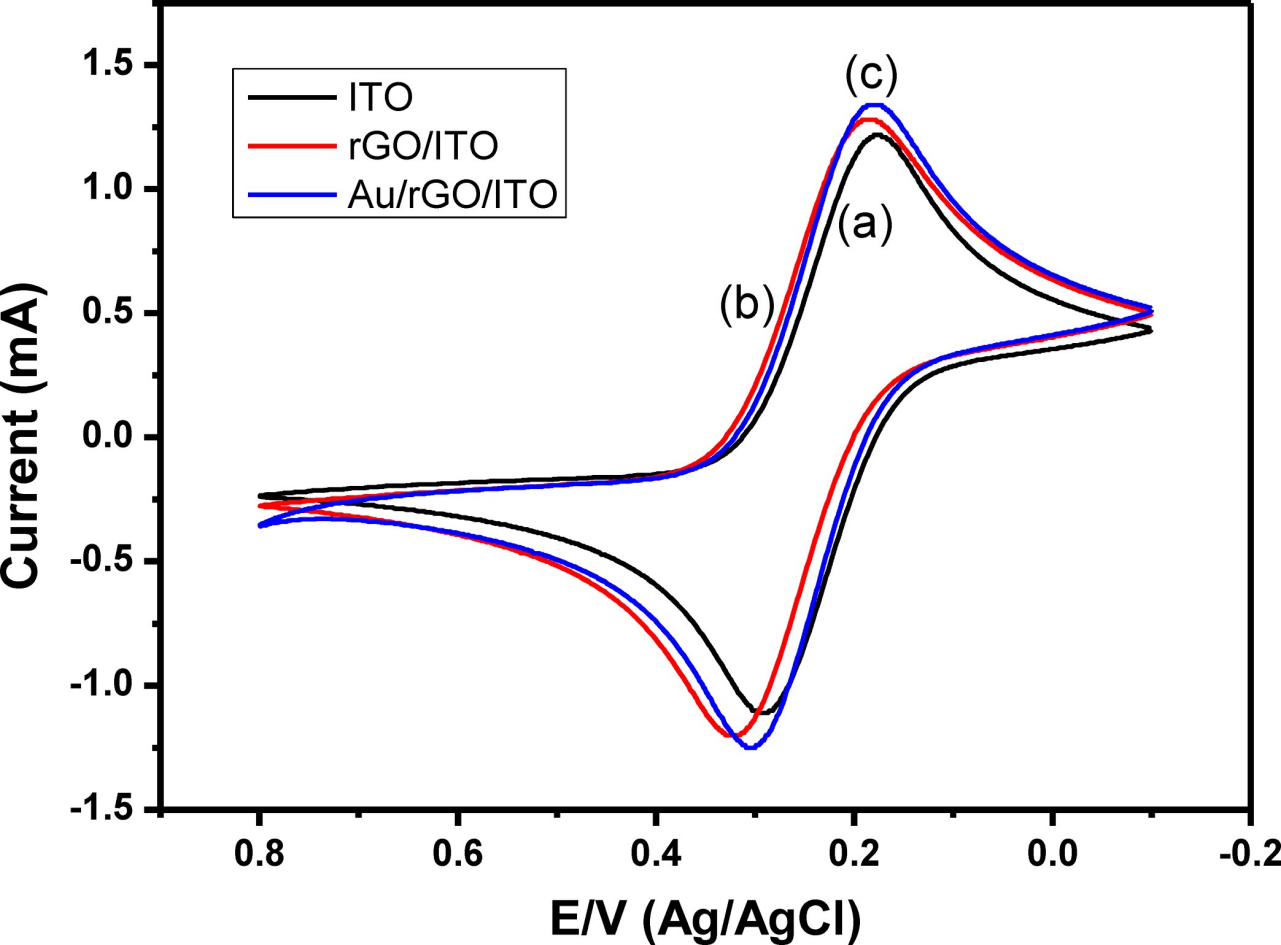
Cyclic voltammograms of bare ITO (curve a), rGO/ITO (curve b) and Au/rGO/ITO (curve c) electrodes in 5 mM of [Fe(CN)_6_]^4-/3-^, a scan rate of 100 mV/sec.

The electrochemical activity of the modified electrodes (rGO/ITO and rGO/Au/ITO) in comparing with bare ITO was performed by measuring their cyclic voltammetric response in 5 mM solution of [Fe(CN)_6_]^4-/3-^ at a scan rate of 100 mV/s. **Fig 3** showed the CVs of bare ITO electrode, rGO/ITO electrode, and rGO/Au/ITO electrode in 5mM of Fe(CN)_6_^4-/3-^ solution. The CV of bare ITO electrode in [Fe(CN)_6_]^4-/3-^ demonstrated a reversible redox behavior with an oxidation peak at 0.3 V and a cathodic peak at 0.175 V (**Fig 3, curve a**). The peak currents increase after deposition of rGO onto the ITO surface, which indicates the improvement of the electrochemical conductivity of the modified electrode in comparison with the bare ITO electrode (**Fig 3, curve b**). These results are in an agreement with the previously reported results by Jiang et al. (2016) and Zhang et al., (2013) [19, 20], which reported that the modification of ITO electrode with GO results in enhancing the electrical conductivity of the bare ITO electrode.

Furthermore, deposition of Au nanodots onto rGO nanosheets/ITO electrode results in increasing the magnitude of redox peaks current as shown in **Fig 3, curve c**, which illustrates increasing the electroactive area of the modified electrode that leading to the increased current in contrast to the bare ITO and rGO/ITO electrodes. These results indicate that the electrochemical activity of ITO electrode was improved by electrochemical deposition of rGO-Au hybrids and the faster electronic transfer was obtained.

### 3.3 Electrochemical performance of the designed AFB1 sensor

**Fig 4:**
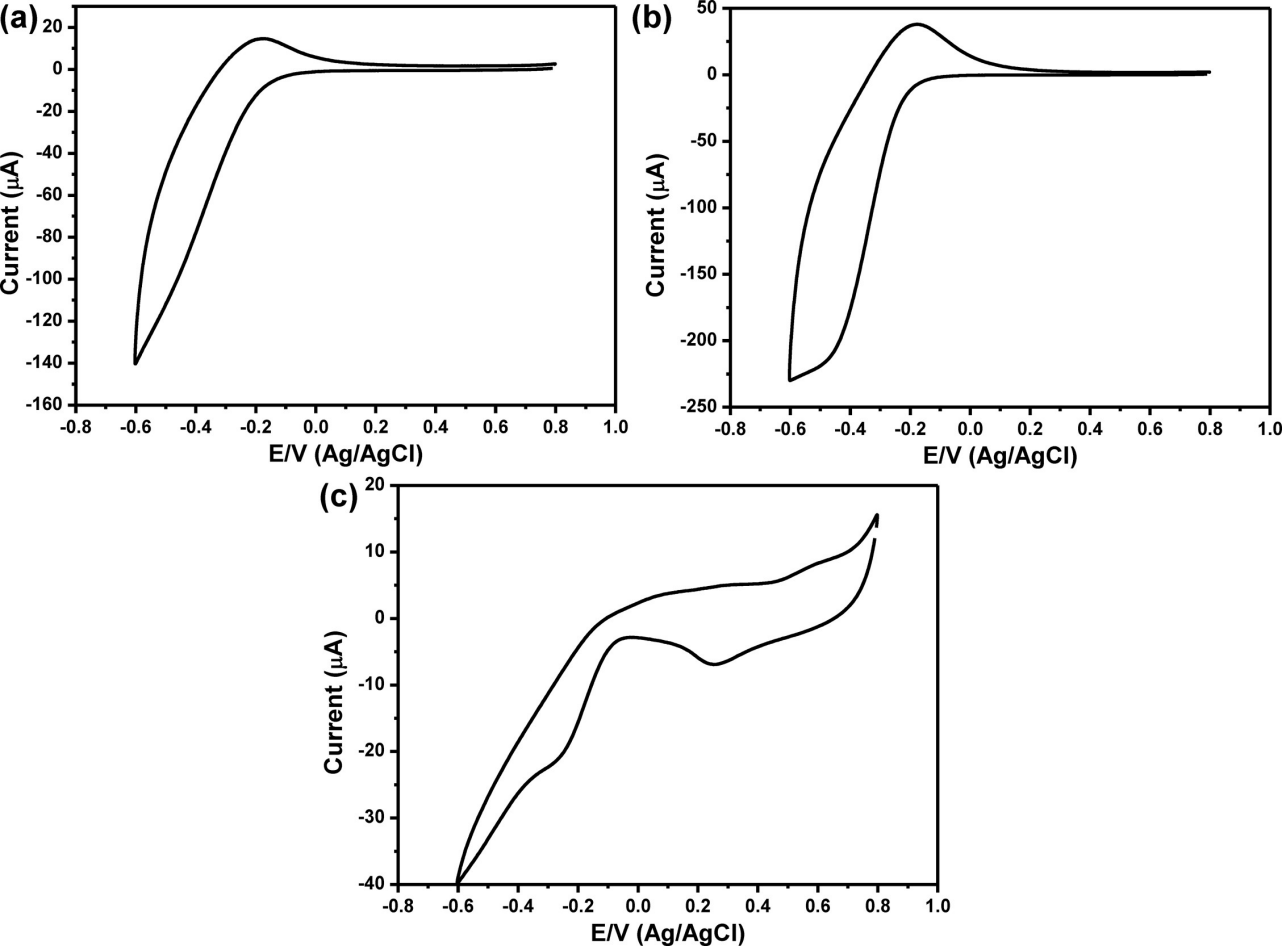
(a) Raman spectrum of anti-AFB1 immobilized onto Au nanodots modified/rGO/ITO electrode, (b) Raman spectra of different concentrations of AFB1 after interaction with anti-AFB1/Au nanodots modified/rGO/ITO electrode, and (c) Intensity of Raman band at 1461 cm^-1^ against AFB1 concentrations.

**Fig 5:**
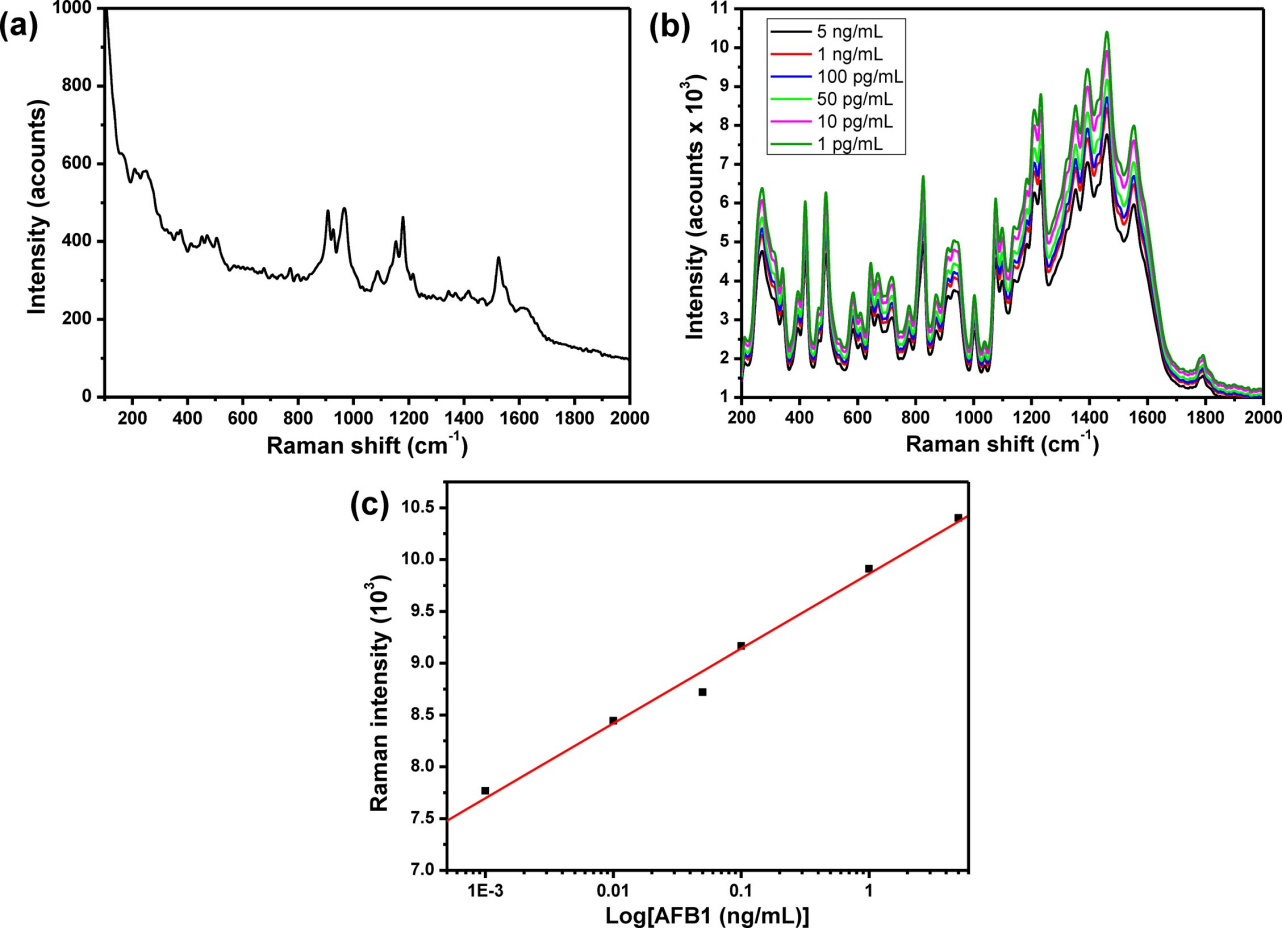
Cyclic voltammetry response of (a) Au nanodots/rGO/ITO, (b) anti-AFB1/Au nanodots/rGO/ITO, and (c) AFB1/anti-AFB1/Au nanodots/rGO/ITO electrodes in 10 mM PBS (pH, 7.4), scan rate 100 mV/sec.

**Fig 6:**
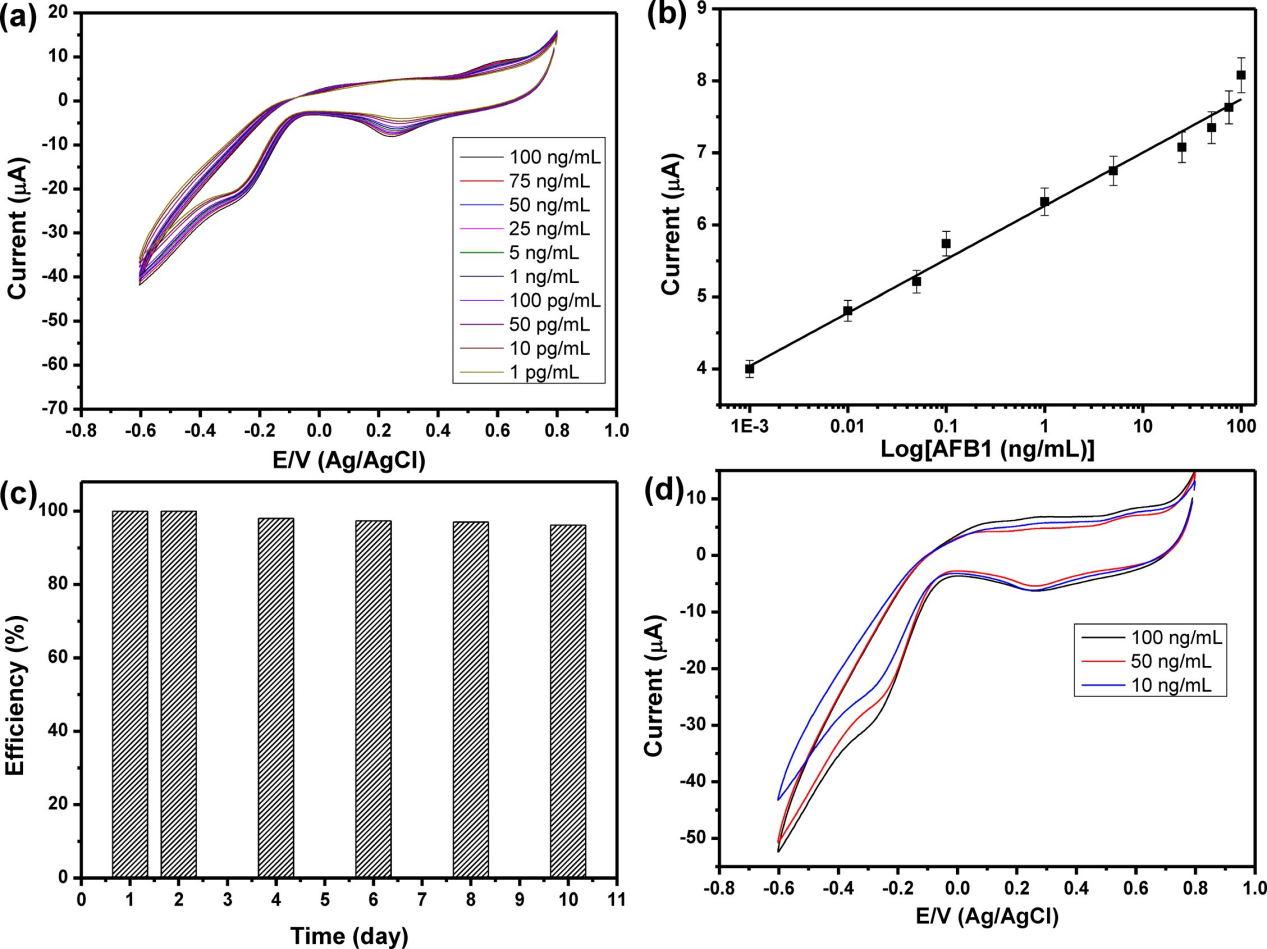
(a) Cyclic voltammograms for different concentrations of AFB1, (b) relationship between the AFB1 concentrations and the cathodic current peaks, the results are the average values of three experiments, (c) the stability of developed sensor toward the detection of AFB1 over 10 days, and (d) cyclic voltammograms for monitoring different concentrations of spiked AFB1. All the measurements were performed at a scan rate of 100 mV/sec in 10 mM PBS (pH, 7.4).

To construct the AFB1 sensor, 10 μg/mL of anti-AFB1 solution was immobilized onto Au nanodots/rGO nanosheets/ITO electrode based on self-assembly technique in which the Au nanodots were acting as anchoring points for anti-AFB1melucolues. The anti-AFB1 antibodies immobilized on the electrode could provide specific binding sites for AFB1 antigens. Immobilization of anti-AFB1 onto Au/rGO/ITO and its interaction with AFB1 was confirmed by using Raman spectroscopy. **Fig 4A** showed the Raman spectrum of anti-AFB1immbolized onto Au/rGO/ITO, which showed a set of Raman bands at about 865, 965, 1212 and 1585 cm^−1^ corresponding to ring deformation, C−O Str., C−H in-plane bending; Str. C−O−C; Str., and C−C Str., respectively. **Fig 4B** showed the Raman spectra of different concentrations of AFB after interaction with anti-AFB1/Au/rGO/ITO. The Raman spectrum showed the major bands that characteristics for AFs including the Raman band at 1461 cm^−1^ (C = C, str. vibration), 935 cm^−1^ (C−O bond) and Raman band at 1551 cm^−1^ that corresponding to str. mode between carbons were observed. Also, the C−H in-plane and out-of-plane bending modes appeared at 686 and 751 cm^−1^. In addition, Raman band at 608 cm^−1^ is related to the ring deformation mode. The assignments of all the Raman bands were summarized in **Table 1 [21]** that confirmed the capability of our sensor to detect AFB1. The intensity of Raman band at 1461 cm^−1^ was used against the AFB1 concentration was represented in **Fig 4C**, which showed a linear relationship within the range from 5 ng/mL to 1 pg/mL. The LOD of AFB1 sensor was calculated from the calibration curve based on 3.3s/S, and it is about 8.1 pg/mL. **Fig 5A** showed the CV behavior of the modified electrode in PBS buffer solution (pH, 7.4), which indicated that the modified electrode didn’t show any redox peaks in PBS solution within the potential range from 0.8V to -0.6 V. The CV of anti-AFB1/Au/rGO/ITO in PBS buffer solution (pH, 7.4) was shown in **Fig 5B**, which demonstrated an irreversible behavior with only one peak at about -0.4 V that can further indicate the successful immobilization of anti-AFB1 onto the modified electrode. To validate the capability of the developed sensor for the detection of AFB1; 100 ng/mL of AFB1 could interact with anti-AFB1 and then the CV response of the antibody/antigen system was detected as shown in **Fig 5C** that illustrated the appearance of a couple of redox peaks at 0.2V and 0.6V, which indicated the capability of the modified electrode for detection of AFB1. To investigate the sensitivity of the developed AFB1 sensor, the electrochemical response of anti-AFB1/Au nanodots/rGO nanosheets/ITO electrode towards a range of AFB1 concentrations from 100 ng/mL to 1 pg/mL were investigated as shown in **Fig 6A**, which demonstrated that the peak current was increased as the concentration of AFB1 increased. **Fig 6B** represented the relationship between the oxidation current peak value and the concentration of AFB1 in which the experiment was repeated three times and the average values were used. Our data illustrated a linear relationship within the range from 100 ng/mL to 1 pg/mL with R^2^ = 0.988 and slop of 0.74. The LOD of AFB1 sensor was calculated from the response curve based on 3.3s/S, and it found to be about 6.9 pg/mL, which is less than the recommended concentration of AFs (0.05 ppm) **[22]**. **Table 2** showing the LOD of the developed sensor in comparing with some of the other electrochemical AFB1 sensors those reported in the literatures **[8, 23–34]**, which confirmed that the developed sensor exhibited a very good LOD in comparing with other sensors.

**Table 1:** SERS spectral assignments for AFB1.

Raman shift (cm^−1^)	band assignments
609	Ring deformation
675	C−H in-plane bending
759.5	C−H out-of-plane bending
827	ring deformation
935	ring breathe, Str. (C−O)
1003	Ring deformation
1038	Str. (CH_2_−CH_2_), β(C-H) ring, β(C-H)-(CH_3_)
1075.5	Str. (C−C−C) ring deformation
1099	Str. C–O–CH_3_ ring deformation
1139	Str. (C−O−CH_2_) (cyclopentene ring)
1184	Out-of-plane bending (C−H) (ring)
1232	In-plane bending (C−H) ring deformation; Str. C−O−C; Str. C−O−C(H_3_)
1320	Asymmetrical (C−H_2_) (ring) (C−H)
1350	Out-of-plane bending CH_3_
1391	Str. CH_3_
1426.5	In-plane bending (C−H) (CH_3_), In-plane bending (C−H) (ring)
1461	Str. (C = C), ring deformation
1551	Str. (C−C) and ring deformation
1794	C = O (2H-pyrane ring)

**Table 2:** Comparison of the limit of detection of the developed sensor with other electrochemical sensors reported in the literatures.

No	Type of electrode	LOD	Reference
**1**	anti-aflatoxin antibodies/gold/quartz crystal	0.012 ng/mL	[8]
**2**	AFB1antibody/ polyaniline modified Pt	100ng/mL	[23]
**3**	polythionine/Au NPs-modified glassy carbon electrode	0.07 ng/mL	[24]
**4**	signal-amplified electrode	0.06 ng/mL	[25]
**5**	acetyl cholinesterase	0.05 μg/ml	[26]
**6**	Au nanorods	0.16 ng/mL	[27]
**7**	GC/polyNeutral Red/ Polycarboxylated thiacalix[4]arene A	0.03 ng/mL	[28]
**8**	GO-Au	0.23 ng/mL	[29]
**9**	Aptamer and DNA probes	0.1 ng/ml	[30]
**10**	Chemiluminescence competitive aptamer	0.11 ng/mL	[31]
**11**	reduced graphene oxide polypyrrole nanocomposite	10 pg/mL	[32]
**12**	anti-aflatoxin antibody on gold electrode	0.5 ng/mL	[33]
**13**	PEDOT/Au-NPs/ITO	4.5 pg/mL	[34]
**14**	anti-aflatoxin antibody/Au/rGO/ITO	6.9 pg/mL	this work

### 3.4. Detection of AFB1 in a real sample

Before studying the efficiency of the developed sensor for monitoring AFB1 in a real sample, the sensor stability was investigated based on storage the sensor at a constant temperature at 4°C and measure its current response. **Fig 6C** showed the efficiency (%) of the developed sensor for detecting 50 ng/mL over ten days; which demonstrated that this sensor could be retained about 96% of its original response after 10 days of storage. To validate the ability of our sensor to detect AFB1 in a real sample, peanut samples were spiked with different concentrations of AFB1 (10, 50 and 100 ng/mL), and their CVs responses were detected as shown in **Fig 6D**, which showed similar behavior like that was represented in **Fig 6B**. Then, their concentrations were calculated by using the values of their cathodic current peaks and calculated the corresponding AFB1 concentrations from by using the calibration curve (**Fig 6C**). The recovery efficiency of the developed sensor was studied and represented in **Table 3** that showed a good recovery percentage from 97.6% to 102.8%. The above results demonstrated the ability of our sensor to monitor the AFB1 in spiked peanut samples and hence it could be used for analysis AFB1 in real samples.

**Table 3:** Recovery percentage of different concentrations of spiked AFB1.

No	Spiked (ng/mL)	Found (ng/mL)	Recovery %
**1**	**100**	**97.6**	**97.6**
**2**	**50**	**49.16**	**98.3**
**3**	**10**	**10.28**	**102.8**

## Conclusions

Here, we have developed a highly sensitive AFB1 sensor for monitoring AFB1 in spiked solution. In this work, we have prepared Au nanodots/rGO nanosheets/ITO electrode based on layer-by-layer electrochemical deposition of Au and rGO, respectively. Morphology and the electrical conductivity of the Au nanodots/rGO nanosheets/ITO electrode were characterized by SEM, and CV behavior of [Fe(CN)_6_]^3-/4-^. The modified electrode was treated with anti-AFB1 and applied as a sensor for AFB1 in PBS based on CV technique, which showed low LOD of about 6.9 pg/mL. Furthermore, the developed sensor was showed good ability to detect AFB1 in spiked samples, which confirm the capability of the developed for real sample sensing applications.

## Supporting information

S1 Data Minimal data set. (XLSX)
